# Cultural significance of wild mammals in mayan and mestizo communities of the Lacandon Rainforest, Chiapas, Mexico

**DOI:** 10.1186/s13002-015-0021-7

**Published:** 2015-05-07

**Authors:** Yasminda García del Valle, Eduardo J Naranjo, Javier Caballero, Carlos Martorell, Felipe Ruan-Soto, Paula L Enríquez

**Affiliations:** Instituto de Biología, Universidad Nacional Autónoma de México, México, DF Mexico; El Colegio de la Frontera Sur. San Cristóbal de Las Casas, Chiapas, Mexico; Departamento de Ecología y Recursos Naturales. Facultad de Ciencias, Universidad Nacional Autónoma de México, México, DF Mexico; Instituto de Ciencias Biológicas, Universidad de Ciencias y Artes de Chiapas, Tuxtla Gutiérrez, Mexico

**Keywords:** Ethnobiology, Ethnozoology, Uses of fauna, Lacandon rainforest

## Abstract

**Background:**

Several ethnobiology studies evaluate the cultural significance (CS) of plants and mushrooms. However, this is not the case for mammals. It is important to make studies of CS allowing the comparison of cultural groups because the value given to groups of organisms may be based on different criteria. Such information would be valuable for wildlife preservation plans. In this study, the most culturally significant species of mammals from the Lacandon Rainforest (Chiapas, Mexico) for people from two Mayan-Lacandon and mestizo communities were identified. The reasons behind the CS of the studied species were explored and the existence of differences among the cultural groups was evaluated.

**Methods:**

One hundred ninety-eight semi-structured and structured interviews were applied to compile socio-demographic information, qualitative data on CS categories, and free listings. Frequency of mention was a relative indicator to evaluate the CS of each species of mammal. Comparison of responses between communities was carried out through multivariate analyses. The non-parametric Mann–Whitney *U* test was used to compare the number of mentioned species by Lacandons and mestizos as well as different responses in the qualitative categories. A *χ*2 test was used to compare frequency of categories.

**Results:**

38 wild mammal species were identified. The classification and Principal Components Analyses show an apparent separation between Lacandon and mestizo sites based on the relative importance of species. All four communities mentioned the lowland paca the most, followed by peccary, white-tailed deer, armadillo, and jaguar. No significant difference was found in the number of mentioned species between the two groups. Eight CS categories were identified. The most important category was “harmful mammals”, which included 28 species. Other relevant categories were edible, medicinal, and appearing in narratives.

**Conclusions:**

The data obtained in this study demonstrates the existence of differential cultural patterns in the relationships that Lacandon and mestizo groups establish with mammals. Species are deemed important either because they are eaten of because of the harm they cause. We suggest the incorporation of local conceptions about wild animals in conservation frameworks for the fauna in the Lacandon Rainforest.

## Resumen

**Introducción:**

Diversos estudios en etnobiología evalúan la importancia cultural (IC) de especies de plantas y hongos. Sin embargo, este no es el caso para mamíferos. Es importante realizar estudios sobre IC que permitan comparar información entre culturas, ya que la valoración de organismos se basa en diferentes criterios. Tal información será útil en planeación para la conservación de vida silvestre. En este estudio se identificaron las especies de mamíferos de mayor importancia cultural para dos comunidades mestizas y dos maya-lacandonas de la Selva Lacandona de Chiapas, México. Se exploran las razones de tal importancia y se evalúan las diferencias entre ambos grupos.

**Métodos:**

Se realizaron 198 entrevistas semiestructuradas y estructuradas para recabar información sociodemográfica, datos cualitativos de categorías de importancia cultural y listados libres. Se utilizó la frecuencia de mención como indicador relativo para evaluar la importancia cultural de cada especie. Las comparaciones de respuestas se realizaron con análisis multivariados. Para comparar el número de especies mencionadas entre grupos y categorías se realizaron pruebas no paramétricas U de Mann Whitney y se realizaron pruebas de *χ*2 para comparar la frecuencia de categorías.

**Resultados:**

Se identificaron 38 especies de mamíferos silvestres. Los análisis de clasificación y componentes principales muestran una aparente separación entre poblados lacandones y mestizos con base en la importancia relativa de las especies. Para las cuatro comunidades la especie más mencionada fue el tepezcuintle, seguido del puerco de monte, el venado cola blanca, el armadillo y el jaguar. No existió diferencia significativa en el número de especies mencionadas entre las poblaciones. Se registraron ocho categorías de importancia cultural. La categoría más importante fue “mamíferos dañinos” con 28 especies, seguido de comestibles, medicinales y con presencia en las narrativas.

**Discusión:**

Nuestros datos muestran que existen patrones culturales diferenciales en las relaciones establecidas entre grupos lacandones y mestizos con los mamíferos. Las especies son consideradas importantes tanto por el aprovechamiento que hacen de ellas como por el daño que pueden ocasionar. Se sugiere incorporar las concepciones locales de mestizos y lacandones sobre los mamíferos silvestres a esquemas y estrategias de conservación de fauna en la Selva Lacandona.

## Background

Human beings order the universe around them to understand it and place themselves in it [1]. Consequently, each human group has developed its own outlines for the taxonomy and classification of biodiversity.

However, no cultural group has named all the elements in nature; this action far exceeds the capability of local taxonomic systems [2]. People give detailed names and classify only those organisms which have a certain degree of proximity to the human domain [3,4], that is, those which are culturally significant. This cultural significance (CS) is given either by the status of “useful resource” or some other interest for a given human group [1]. The concept of cultural significance arose through the study of traditional systems of taxonomy and classification. Hunn [2] defined the cultural significance of a taxon as the value of the role it carries out within a culture.

Rural communities make use of a great host of available resources; however, they are not all equally valuable. There are preferences of certain species or groups of species [5,6]. These culturally salient organisms exhibit a wide range of importance, that is, in they include both species of extreme relevance and species with minimum significance [7]. In this sense, the valuation each culture makes of elements of nature depends on diverse reasons [8] both extrinsic and intrinsic. The conception of a species, its particular ecological features, the benefits generated by its use (food, medicine, raw material), the direct or indirect harm it can cause, its commercial, symbolic, and spiritual value, and other criteria, are examples of tangible and intangible features that communities take into account to assign value [9]. Said valuation involves different social and ecological processes which are particular to each population and happen in a different fashion through time. Thus, the cultural significance of a plant or animal is an eminently historical process [10].

A number of studies have aimed to comparatively estimate the CS of plants and mushrooms [11-15]. However, for fauna studies such estimates are scarce [16,17] since the tendency has been to evaluate hunting [18], zootherapy [19], and quantifying their use value [20], while little attention has been paid to quantifying their cultural significance.

Fauna resources have been of importance in many diverse aspects of human life from its beginnings. The relation between animals and humans far surpasses utilitarian aspects; animals are present in religion, art, music, literature, and many other human manifestations [21]. Thus, to understand this relationship, ethnozoology should consider an affective domain [22] and take the cultural and social bonds between local communities and these organisms into account [17]. Mammals in particular have been considered one of the most important groups for several reasons: a) in many communities they constitute the main source of animal protein because of their size and the high probability of obtaining an energetic surplus if hunted, b) they are used in zootherapy, to make clothes and tools, and c) they have a central role in mythology [18,21].

The problem ethnobiology, and ethnozoology in particular, faces is the documentation of the level of significance of a particular taxon and the distinction of more important organisms and the reasons for this differential relevance [7]. Since the seventies, numerous ways to evaluate the level of significance through a quantitative focus have been put forward [7].

Among the most popular techniques are indexes based on informant consensus –defined as the degree of agreement among the interviewed about a given resource [14,23]. These indexes are based on the premise that the more important an organism is for a community, the more likely it is to be named. The preferred indicators of this are the frequency and order of mention [15,24]. The elements which are most frequently mentioned, and those mentioned first during the interviews are assumed to be those of greater CS for the studied population [25,26]. This procedure tends to be more impartial, given that it is designed to minimize the bias the investigators, who may relate the CS of an organism to certain indicators and/or features according to their own prejudice –*etic* design of the index– [27]. Nonetheless, order and frequency of mention also have limitations because sometimes the most mentioned organisms are not those which are currently most useful [14]. Furthermore, these indicators give no clue as to why people assign a given valuation to each element.

On the other hand, different authors have pointed out the need to carry out resource CS evaluations through techniques which allow comparisons among different cultures [7,11]. This is due to the fact that communities value organisms according to dissimilar criteria which reflect value systems unlike those of occidental societies [23]. Differences in worldview–understood as the way people explain the origin and order of the universe and the way humans are to behave in it [28]–generate completely contrasting attitudes towards land and natural resources. Comparison between indigenous and mestizo (non-indigenous) groups, which in general have distinct worldviews even if they share a region with similar resources, constitutes a unique opportunity for the study of cultural significance.

Understanding how different cultural groups value their resources, which species are considered the most important, and, above all, the reasons behind this generates valuable information for decision making concerning fauna, and particularly so for mammals, conservation. It can be expected that people are more motivated to preserve significant resources than less important species [23]. Thus, it is inconceivable that mammal conservation strategies fail to take the relationship between communities and mammal fauna into account [21]. This makes the importance of ethnozoological studies evident.

In this paper the aim is recognizing the most significant wild mammal species for the people in four communities of the Lacandon Rainforest, Chiapas, Mexico. Two of these communities are Lacandon Mayan and two are mestizo. CS is evaluated and the hypothesis that both species composition and valuation are different in cultures with dissimilar traditions is tested.

## Methods

### Study site

**Figure 1:**
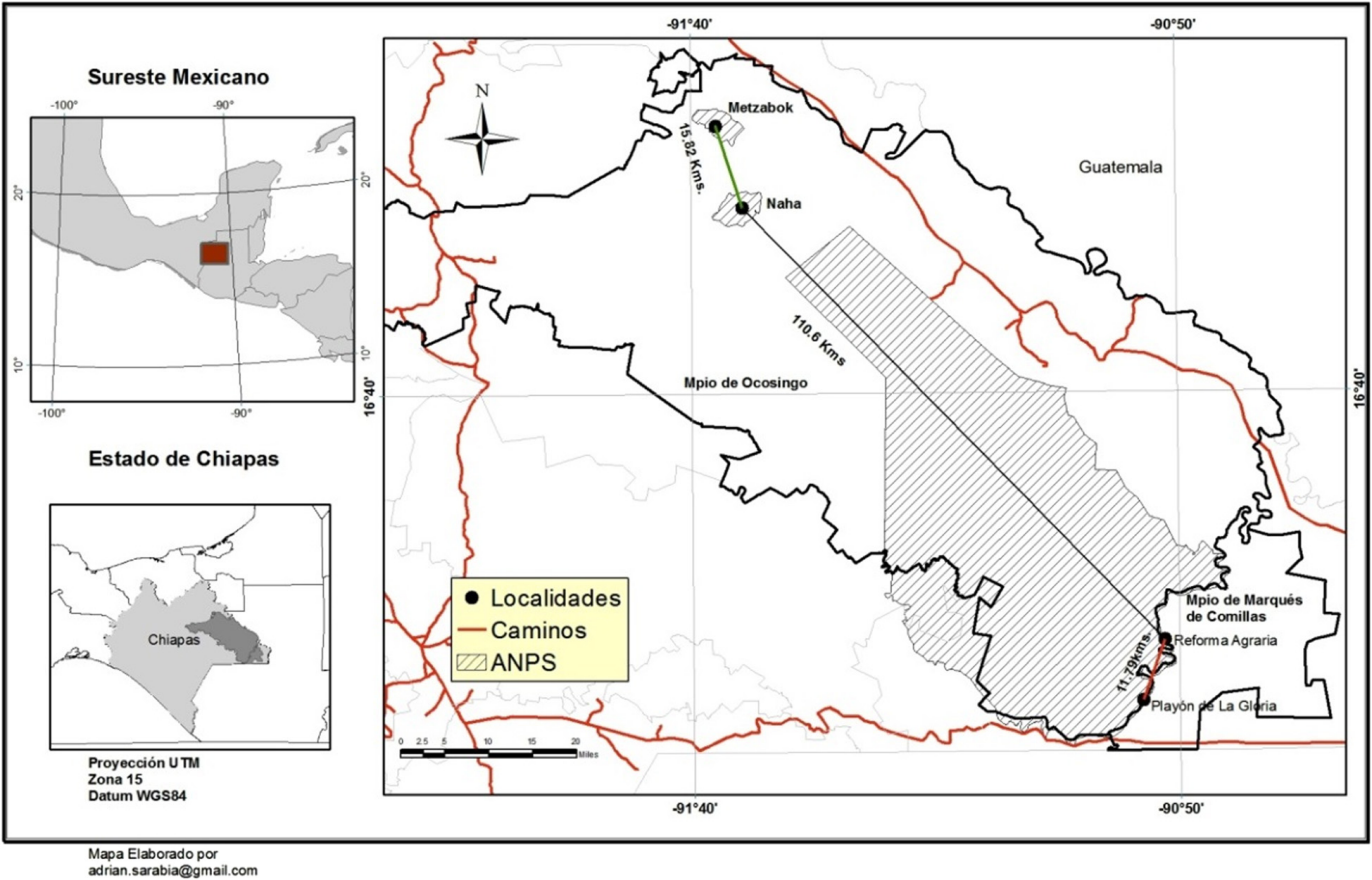
Location of Naha, Metzabok, Playon de la Gloria, and Reforma Agraria, Chiapas, Mexico.

The Lacandon Rainforest is located in the East-Northeast region of the state of Chiapas (Figure 1). The prevailing climate is warm-humid (23-27°C). Altitude varies from 10 to 900 MASL. Predominant vegetation is highland rainforest, although pine forests are found in the higher zones [29]. The Lacandon Mayan communities of Naha and Metzabok were decreed an Area for the Protection of Flora and Fauna (APFF) in 1998. Both communities have highland and midland rainforest vegetation with patches of cloud forests and pine and oak forests, as well as secondary vegetation, maize fields, and vegetable cultivars. Naha has 198 inhabitants grouped in 46 families and Metzabok has a population of 96 inhabitants grouped in 20 families. The main economic activities in both communities are agriculture and tourism [30], though Naha receives greater resources for this activity than does Metzabok, where tourists arrive to a lesser scale. The mestizo communities of the common lands of Playon de la Gloria and Reforma Agraria are adjacent to the Biosphere Reserve Montes Azules. They have highland and midland rainforests, as well as secondary vegetation in diverse stages of regeneration and lands dedicated to crop growing and stockbreeding. Playon de la Gloria has approximately 209 people grouped in 44 families and Reforma Agraria has 145 people grouped in 30 families. Both common lands are mainly inhabited by stockbreeders, farmers, and people dedicated to tourism [29,30]. In this case, Reforma Agraria receives greater benefits from tourism than does Playon de la Gloria, where this is an infrequent activity.

### Data collection and analysis

Before fieldwork, informed consent was obtained from the common lands authorities and the environmental authorities in all four communities for the publication of any data and images collected throughout this research. From January to October, 2013, 189 semistructured and structured interviews [31] were carried out with randomly selected people (55 in Naha, 32 in Metzabok, 52 in Playon de la Gloria and 50 in Reforma Agraria). The semi-structured interview covered the topics of local taxonomy and systematics, conceptions about mammals, worldview aspects, ecological knowledge, management practices and use of mammals, economic aspects, and knowledge transmission. The structured interview consisted of a set of social-demographic questions, a free listing of known wild mammals, and a taxonomic corroboration exercise of local names. For this, a field guide with photographs of the registered mammals in the Lacandon Rainforest was designed.

Frequency of mention was used as an indicator of CS of wild mammals for the studied populations. Thus, the most mentioned mammal in the interviews was considered the most important [15,24,32]. Semi-structured interviews were analyzed by constant comparison of analysis categories as suggested by Sandoval [33]. To explore the differences between the studied communities based on the relative frequency of mention of mammal species, a matrix of distances was calculated through the average taxonomic distance method. These values were subject to a cluster analysis –following the Unweighted Pair Group Method with Arithmetic Mean (UPGMA) method– and a Principal Components Analysis (PCA) using the 2.11 version of NTSYS (Numerical Taxonomy and Multivariate Analysis System) for PC [34]. These analyses helped explore the patterns of variation in the responses of the free listings. To determine significant differences between mestizo and Lacandon population in number of mentioned species in the free listings and number of mentioned species per CS category, a Mann–Whitney test was carried out. Finally, a *χ*^2^ test was used to compare the frequency of categories.

## Results

The total number of recognized wild mammals by the people in all four study sites was 38 local taxa. In general, these taxa are classified within 10 orders, 20 families, and 30 genera (Table 1). In each pair of communities–Lacandon and mestizo–, the number of recognized species was 35 (34 in Naha and 33 in Metzabok; 33 in Playon de la Gloria and 32 in Reforma Agraria).

**Table 1:** **Locally recognized mammal species, scientific names, names in Lacandon Maya, frequency of mention, and use categories mentioned in the communities Playon de la Gloria, Reforma Agraria, Naha, and Metzabok, Chiapas, Mexico**

	**Local species**	**Scientific name**	**Maya-Lacandon name**	**Use categories**	**Total Fr/Me**	**Mest.Fr/Me**	**Lac.Fr/Me**
1	Tepezcuintle	*Cuniculus paca*	Haré	Co, Da, Na y Ma	163	82	81
2	Puerco de monte	Tayassuidae Family	Hax kekan	Co, Da, Me y Ma	157	88	69
3	Venado cola blanca	*Odocoileus virginianus*	Ké	Co, Da, Me, Na, Ar, UH, Or y Ma	148	84	64
4	Armadillo	*Dasypus novemcinctus*	Huech	Co, Da, Me, Na y UH	124	75	49
5	Jaguar	*Panthera onca*	Hax barum	Co, Da, Me, Na y Or	105	58	47
6	Tlacuache	Didelphidae Family	Kan’ och	Da y Me	99	50	49
7	Tejon	*Nasua narica*	Sú sú	Co, Da, Me y Na	95	47	48
8	Mapache	*Procyon lotor*	A’ka’bak	Co, Da y Ma	88	55	33
9	Saraguato	*Alouatta pigra*	Ba’st	Co, Me y Na	82	47	35
10	Tapir	*Tapirus bairdii*	Caxitzimin	Da, Me y Na	77	63	14
11	Mono araña	*Ateles geoffroyi*	Ma’ax	Co, Me, Na y Ma	74	45	29
12	Ardilla	Sciuridae Family	Ak’ kuk	Co, Da y Me	64	31	33
13	Sereque	*Dasyprocta punctata*	Tzub	Co, Me y Or	59	15	44
14	Tigrillo	*Leopardus wiedii*	Mam bore’	Da, Na y Or	52	33	19
15	Zorrillo	Mephitidae Family	Apay	Co, Da y Me	47	20	27
16	Venado cabrito	*Mazama temama*	Yuk	Co, Da, Ar, UH y Or	43	25	18
17	Tuza	Geomyidae Family	Baá	Co, Da, Me y Na	39	16	23
18	Martucha	*Potos flavus*	Ak max	NC	37	19	18
19	Oso hormiguero	*Tamandua mexicana*	Aj chap’	Da	30	16	14
20	Zorra gris	*Urocyon cinereoargenteus*	Chámak	Da	30	10	20
21	Leoncillo	*Puma yagouaroundi*	Ek barum	Da	27	20	7
22	Nutria	*Lontra longicaudis*	Tzurei ha	Na	26	15	11
23	Puma	*Puma concolor*	Chaak barum	Da y Or	25	15	10
24	Raton	Muridae Family	Chok	Da	24	14	10
25	Conejo	Sylvilagus Family	At tuur	Co	22	9	13
26	Puerco espin	*Sphiggurus mexicanus*	Kix pach	Da y Na	21	10	11
27	Cabeza de viejo	*Eira barbara*	Sanjor	Da	18	15	3
28	Ocelote	*Leopardus pardalis*	Ek xux	Da	12	6	6
29	Murcielago	Chiroptera	Sek	Da y Na	12	4	8
30	Cacomixtle	*Bassariscus sumichrasti*	Ha yuk	NC	6	0	6
31	Armadillo de cola desnuda	*Cabassous centralis*	Kitam huech	Da	5	0	5
32	Coyote	*Canis lantras*	Peki cash	Da	4	1	3
33	Comadreja	*Mustela frenata*	Ag sabin	NC	4	3	1
34	Mico dorado	*Cyclopes didactylus*	Cha’ak chap’	Na	3	3	1
35	Raton tlacuache	*Marmosa mexicana*	Chok och*	Da	3	3	0
36	Tlacuache acuático	*Chironectes minimus*	Han och*	Da	2	2	0
37	Grison	*Galictis vittata*	Sanjor*	Da	1	1	0
38	Tlacuache dorado	*Caluromys derbianus*	Zek tu biix	NC	1	1	1

**Total Fr/Me =** total frequency of mention, **Mest. Fr/Me =** frequency of mention in mestizo communities, **Lac.Fr/Me =** frequency of mention in Lacandon communities, **Co =** Edible, **Da =** Harmful, **Me =** Medicinal, **Na =** Narratives, **Ma =** Pet, **Ar =** Artisan use, **Or =** Ornamental use, **UH =** Utensils and/or tools, and **NC =** uncategorized. *Names mentioned in interviews with the Lacandon participants after the free listings.

A high percentage (78%) of the local taxa the interviewed identifies corresponds to a single taxonomic species (e.g. *tepezcuintle* –paca–corresponds to the taxon *Cuniculus paca*). However, eight of the identified local taxa do not correspond to scientific taxa, but rather are included in taxonomic groups of higher hierarchy (Table 1). For example, the local taxon *puerco de monte* (peccary) includes two species: *Pecari tajacu* y *Tayassu pecari.* In other cases, the taxón *zorrillo* (skunk) includes four species of the Mephitidae family or the local taxon *raton* (mouse) includes all mice species in the region. Berlin *et al.* [35] call these correspondences one to one relations and sub-differentiation in an attempt to establish correlations between folk taxonomy systems and Linnean taxonomy.

Five species are recognized by more than 50% of the population (*tepezcuintle, puerco de monte, venado cola blanca, armadillo* and *jaguar –*lowland paca, peccary, white-tailed deer, armadillo, and jaguar respectively–). Meanwhile, 24 species are recognized only by 26% of the population (Table 1). For the interviewed people in all four communities, the most mentioned species was the *tepezcuintle* (86% of all the interviewed) followed by the *puerco de monte* (83%), the *venado cola blanca* (78%), the *armadillo* (65%), and the *jaguar* (55%). The largest number of species mentioned in an interview were 28 and the smallest was one, the average number of cited species was 9.5 (Table 1).

**Figure 2:**
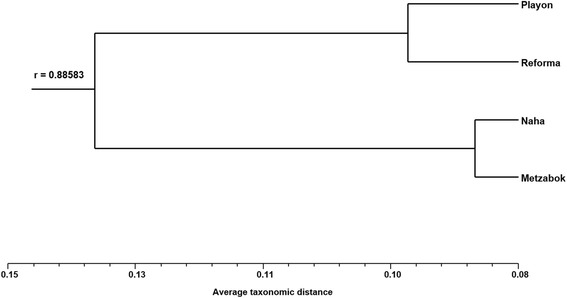
Cluster analysis of the four study sites using the Average Taxonomic Distance index.

**Figure 3:**
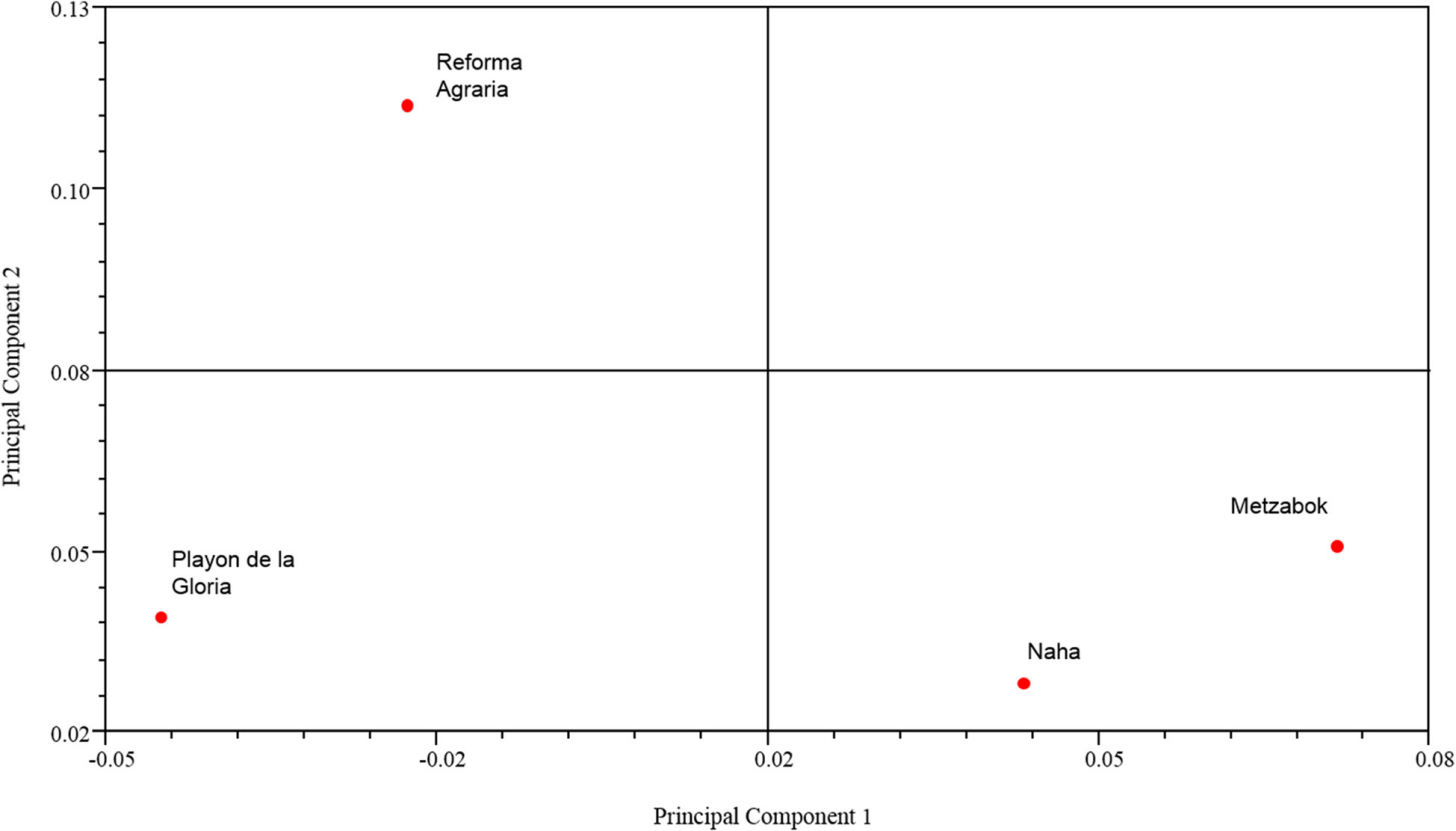
Principal components analysis for the studied communities.

The classification analysis based on the relative frequency of mention of mammal species shows a variation pattern between communities which relates to their cultural traditions. The two Lacandon communities (Naha and Metzabok) are grouped together, as are the mestizo communities (Reforma Agraria and Playon de la Gloria) (Figure 2). The principal components analysis shows that the principal component one explains 67.19% of the variations, separating the Lacandon communities, Metzabok and Naha, from the mestizo communities. The characters with the largest weight are the frequency mentions of the *sereque* (Central-american agouti: *Dasyprocta punctata*), the *cabeza de viejo* (greyheaded tayra: *Eira barbara*), and the *tuza* (gopher: Geomyidae Family). The principal component two, which explains 23.99% of the variation, isolates Reforma Agraria from the other communities. The characters with greatest weights for this are the frequencies of mention for the *tejon* (white-nosed coati: *Nasua narica*), the *saraguato* (howler monkey: *Alouatta pigra*), the *raton* (mouse), and the *nutria* (otter: *Lontra longicaudis*) (Figure 3).

Four species –*tepezcuintle (*paca), *puerco de monte* (peccary), *venado* (deer), and *armadillo–* are the most mentioned by both Lacandons and mestizos, along with *tlacuache* (opossum) in the case of Lacandons, and *tapir* in the case of mestizos. About 84% of the species were mentioned by both groups. However the *grison* (greater grison: *Galactis vittata*), the *raton tlacuache* (*Marmosa mexicana*), and the *tlacuache acuatico* (*Chironectes minimus*) were mentioned exclusively by mestizos, while the *cacomixtle* (*Bassariscus sumichrasti*), the *armadillo cola desnuda* (naked-tailed armadillo: *Cabassous centralis*), and the *tlacuache dorado* (Derby’s wooly opossum: *Caluromys derbianus*) were mentioned only by Lacandons.

There was no significant difference in the number of species mentioned by the Lacandon and mestizo communities according to the Mann–Whitney test (*P =* 0.7450) (Table 2).

**Table 2:** **Number of species mentioned in free listings by the interviewed population from Playon de la Gloria, Reforma Agraria, Naha, and Metzabok, Chiapas, Mexico**

	**Minimum**	**Maximum**	**Average**	**Standard deviation**	**Median**	**Mann–Whitney test**
Mestizos	1	24	9.7	4.9	8	*P* = 0.7450 > 0.05
Lacandons	2	24	9.5	4.9	9	

There were significant differences in the number of times mestizos and Lacandons mention certain species (*χ*2 = 100.91, *P* < 0.001). The tapir, white-tailed deer, peccary, and jaguar are the most mentioned by mestizos (63%, 13.5%, 12.5%, and 10.5% respectively). Contrastingly, the *sereque* (agouti) is mentioned 49% more frequently by Lacandons.

To determine which species are responsible for the differences between the two groups, an analysis of adjusted residuals as suggested by Haberman was carried out (Table 3). Among the species with high frequency of mention, significant differences were found between Lacandons and mestizos in several instances. The *tapir* and *cabeza de viejo* were more frequently mentioned by mestizos; meanwhile, Lacandons mentioned the *sereque*, *zorra gris* (grey fox: *Urocyon cinereoargenteus*), *cacomixtle,* and *armadillo cola desnuda* more frequently.

**Table 3:** **Haberman adjusted residues (significant values are in italics)**

	**Mestizos**	**Lacandons**
Tepezcuintle	1.22	−1.22
Puerco de monte	−0.38	0.38
Venado cola blanca	−0.55	0.55
Armadillo	−1.39	1.39
Jaguar	−0.12	0.12
Tlacuache	0.88	−0.88
Tejon	1.07	−1.07
Mapache	−1.55	1.55
Saraguato	−0.50	0.50
Tapir	*−4.98*	*4.98*
Mono araña	−1.10	1.10
Ardilla	1.04	−1.04
Sereque	*4.65*	*−4.65*
Tigrillo	−1.31	1.31
Zorrillo	1.71	−1.71
Venado cabrito	−0.47	0.47
Tuza	1.75	−1.75
Martucha	0.41	−0.41
Oso hormiguero	0.15	−0.15
Zorra gris	*2.38*	−2.38
Leoncillo	−2.05	2.05
Nutria	−0.31	0.31
Puma	−0.54	0.54
Raton	−0.36	0.36
Conejo	1.31	−1.31
Puerco espin	0.66	−0.66
Cabeza de viejo	*−2.47*	2.47
Ocelote	0.33	−0.33
Murciélago	1.49	−1.49
Cacomixtle	*2.70*	−2.70
Armadillo de cola desnuda	*2.46*	−2.46
Coyote	1.19	−1.19
Comadreja	−0.82	0.82
Mico dorado	−0.82	0.82
Raton tlacuache	−1.58	1.58
Others	−1.14	1.14

The 38 taxa mentioned by the interviewed population are related to eight CS categories: edible, harmful, medicinal, ornamental, used in craft-making, used as utensils or tools, pets, and mammals present in local narratives such as cosmogony myths, histories, or legends (Table 1).

Species that are considered edible are those which are used or have been used as food. Those within the “harmful” category are those which affect the cattle, backyard animals, crops, or the people themselves. Those considered medicinal are species with properties useful to combat disease. “Pets” are species which are kept in the houses, either in confinement or in the backyard, and are considered companion animals. Species considered as utensils or tools are those used in the crafting of everyday useful artifacts such as threshing utensils, bags, or others. Species used as ornaments are those which adorn the house or are considered a luxury. Those used in crafts-making are those whose parts are used to make merchandisable products. Finally, species within the narrative category are those which appear in cosmogonic myths or other tales which are part of the worldview of the cultural group.

The category with the most species is “harmful mammals”, followed by edible mammals, medicinal mammals, and mammals in narrative (Table 4). 84% of the significant species recognized in the four study sites are either edible or harmful. Only the *martucha* (kinkajou: *Potos flavus*), the *cacomixtle*, the *comadreja* (long-tailed weasel: *Mustela frenata*), and the *tlacuache dorado* are not included in any of the CS categories.

**Table 4:** **Number of recognized mammal species in each significance category in Playon de la Gloria, Reforma Agraria, Naha, and Metzabok, Chiapas, Mexico**

	**Accum Tot. Playon de la Gloria**	**Accum. Tot. Reforma Agraria**	**Accum.Tot. Naha**	**Accum. Tot. Metzabok**	**General accum. total**	**Accum total Mest.**	**Ave. Mest.**	**Max. - Min. Mest.**	**Accum Total Lac.**	**Ave. Lac.**	**Max. - Min. Lac**	**Mann–Whitney test**
Edible	11	6	12	11	15	11	2.0	0-10	15	2.1	0-15	*P* = 0.7623 > 0.05
Harmful	26	16	16	14	28	25	0.6	0-8	16	0.7	0-8	*P* = 0.8417 > 0.05
Medicinal	7	6	6	8	14	10	0.2	0-2	9	0.1	0-4	*P* = 0.2505 > 0.05
Narrative	4	4	10	8	14	6	0.1	0-3	12	0.3	0-4	*P* = 0.0506 > 0.05
Artisan use	0	1	1	0	2	1	0.009	0-1	1	0.011	0-1	*P* = 0.9106 > 0.05
Utensils/Tools	3	3	0	0	3	3	0.01	0-2	0	0.00	0-0	--
Ornamental	4	2	4	0	7	5	0.02	0-2	4	0.02	0-2	*P* = 0.6722 > 0.05
Pet	3	2	1	1	5	4	0.04	0-3	1	0.03	0-1	*P* = 0.6633 > 0.05

**Accum. Tot. Playon de la Gloria =** Accumulated total number of recognized species in Playon de la Gloria; **Accum. Tot. Reforma Agraria =** Accumulated total number of recognized species in Reforma Agraria. **Accum. Tot. Naha =** Accumulated total number of recognized species in Naha. **Accum. Tot. Metzabok =** Accumulated total number of recognized species in Metzabok. **General accum. total =** Accumulated total number of recognized species in all four communities. **Accum. Total Mest. =** accumulated total for mestizos. **Ave. Mest. =** average of species mentioned per interviewed individual in mestizo communities. **Max-Min Mest. =** Maximum and minimum of recognized species per interviewed individual in mestizo communities. **Accum. Total Lac. =** accumulated total for Lacandons. **Ave. Lac. =** average of species mentioned per interviewed individual in Lacandon communities. **Max-Min Mest. =** Maximum and minimum of recognized species per interviewed individual in Lacandon communities. **Mann–Whitney test =** test to prove whether there were significant differences in the number of recognized species by mestizos and Lacandons.

Even though there are no significant differences in the number of species included in the categories, some trends are noticeable: in Lacandon communities more species are recognized as edible as are those included in locally transmitted narratives. On the other hand, in mestizo communities, more species are considered harmful, used as pets, and used in the crafting of tools and utensils (Table 4).

The species which were included in more different categories were the *venado cola blanca*–eight categories–, the *armadillo*, the *venado cabrito* (Central-american red brocket: (*Mazama temama*) and the *jaguar*–five categories each–. Four species (*martucha, cacomixtle, comadreja,* and *tlacuache dorado*) were not mentioned in any of the categories.

## Discussion

The number of species (38) recognized by the communities in this study represents 44.46% of the reported mammal fauna of the Lacandon Rainforest [36]. This indicates the importance of this taxonomic group for the studied population. In general indigenous and rural communities of the Neotropic recognize an elevated portion of mammals present in their land as significant. For example, other indigenous and mestizo groups in the Lacandon Rainforest recognize 31 species of mammals [37], while in two communities of Hueytamalco, Puebla, Mexico 36 species are recognized [38], the Shuar in Ecuador recognize 16 species, the Tacana in Bolivia 43, and the inhabitants of Calera, Colombia 19 [39-41].

On the other hand, it is possible to document the level of significance of mammals in the study zone through linguistic analysis and local taxonomy and classification systems. According to Turner [7] the most culturally significant organisms are those with simple, non-analyzable names. 68% of the Spanish names, and 73% of the Lacandon Maya names meet this criterion. Furthermore, most of the significant local species of mammals (78%) have a one-to-one correspondence with biological species as described by Berlin *et al.* [35]. This is, each local generic taxon relates to a single species of the linnean systematics. In the case of bats and mice, for example, there is a sub-differentiation, since one generic name corresponds to all the regional species within the taxonomic group.

Mammals are an extremely charismatic group that is generally present in people’s minds, ethnic condition, cultural traditions, or closeness to wilderness notwithstanding [42]. This is among the reasons we consider might explain why no differences were found in the number of species mentioned in Lacandon and mestizo communities. As mentioned above, mammals have a greater significance than other taxa and a high percentage of local species is recognized. Accordingly, it is not strange that, no matter what their cultural tradition was, the interviewed population mentioned a similar set of species. What is more, both human groups inhabit very similar ecosystems which in all likelihood contain (or recently contained) the same species [43].

Within the four studied communities, *tepezcuintle*, *puerco de monte, venado cola blanca*, and *armadillo* are the most frequently mentioned species. These coincide completely with the species reported in other studies carried out with indigenous and mestizo groups all over the Neotropic region [44-53]. These species are considered important because of their value as food and their contribution to animal protein intake. Moreover, the preference of these mammals based on their flavor and size has been documented [53]. In particular, *tepezcuintle* is considered to carry the best protein value in addition to being considered a tender, tasty, and “clean” meat –meaning it is locally conceived to be free of parasites and harmful substances–. Diverse studies in the Neotropic region single out the *tepezcuintle* as the preferred consumed species [54-59].

In the studied communities, not all of the frequently mentioned species are deemed useful. In addition to the four edible species, the *jaguar* appears among the most mentioned species. While this species was mentioned within three use categories (edible, medicinal, and ornamental), it is also present in non-utilitarian categories, such as “present in narratives” and “harmful species”. The qualitative interviews gathered information supporting that this animal’s harmfulness might be more relevant to the evaluation of its significance than are its uses. Duality is a common feature in Mesoamerican societies’ worldviews [60]; however, the interviewed were always emphatic when pointing out the harmful condition of *jaguar*. This fact is even present within narratives. Among mestizo population this is due to the fact that it sometimes feeds from calves and sheep, even becoming the main reason for cattle disappearance. This is a recurring situation among Neotropic populations engaged in stockbreeding [61-64]. For the Lacandon, jaguars are also considered harmful because they can attack people who wander alone in the wilderness. In Lacandon narratives, the jaguar is conceived as an entity with the capability to transform into a human and deceive Lacandons to bring them into the jungle, get them lost, and eat them. While qualitative interviews shed light on the fact that the “harmful” condition of an animal is a decisive factor on people’s valuation of the jaguar, more precise studies are needed for the evaluation of the quantitative variables of significance (or subindexes) to analyze through multivariate techniques which indicators weigh more in the valuation people makes. Such cultural significance indexes allow a clearer understanding of the reasons behind this phenomenon [13].

Even though the number and composition of mentioned species do not significantly vary between communities, there is evidence of contrasting cultural patterns between the relationships that are established by Lacandon or mestizo groups with the mammals of the Lacandon Rainforest. The classification analyses and PCA show an apparent division between Lacandon and mestizo populations based on the relative significance of the species.

The PCA showed that the characters with the largest weight in the discrimination of the Lacandon and mestizo communities were the frequencies of mention of species such as the *sereque*, *tuza*, and *cabeza de viejo*. The Lacandons stated that both the *tuza* and the *sereque* are abundant and may be frequently observed within crop fields (particularly *milpas*). They are also considered edible. *Tuzas* on the other hand, are considered harmful for crop fields and are mentioned in a cosmogonic myth. On the other hand, for mestizo communities these species are not considered to be neither edible nor abundant.

*Cabeza de viejo* is a species that mestizos are much more aware of than are Lacandons. For both groups, this species is conceived to be harmful, since occasionally it preys on poultry, or other edible animals. Some people even point out it can be aggressive when found in the fields.

Another differential aspect between communities of different ethnic origin is the composition of the lists of the most frequently mentioned mammals. For example, there are species which are exclusively mentioned by one of the groups. *Cacomixtle*, *armadillo de cola desnuda,* and *tlacuache dorado*, for instance, were only mentioned by Lacandons. These species are rare, scarce, nocturnal, and particular to zones with preserved vegetation. These features make them hard to detect for most people, with the exception of nocturnal hunters and occasional viewers. The same stands for the *grison* and *tlacuache de agua*, which have been observed by few in the mestizo communities.

Another evidence of the differences between Lacandons and mestizos concerning their mammal fauna is the number of times each group mentions some of the species. The *tapir, jaguar, venado,* and *puerco de monte* are significantly more mentioned by mestizos, while the *sereque* is more often mentioned by Lacandons. This pattern may be explained by the abundance of these species in different zones of the Lacandon Rainforest. Even though there is no information about mammal abundance for the study areas, there is evidence of local viewings which may be taken into account to state that *tapir*, *jaguar*, *venado,* and *puerco de monte* have a lower or even inexistent abundance in the zone inhabited by the Lacandons. The mestizo communities are located within the buffer zone of the Montes Azules Biosphere Reserve, which encases about 330, 000 Ha of rainforest, an ideal habitat for these species to maintain stable abundances. Given this situation, it is expected for mestizo populations in this area to observe them and interact more or less frequently with them. Contrastingly, in the region inhabited by the Lacandons there are either few registers of species such as *puerco de monte, venado cola blanca, jaguar,* and *tapir* or there have been no registers for several years. Lacandon communites have small rainforest surfaces (around 3000 Ha each) and these are fragmented, surrounded by great areas adapted for stockbreeding from neighboring lands. Even though both territories are Areas for the Protection of Flora and Fauna, habitat reduction and furtive hunting carried out by inhabitants of neighbor communal lands have undoubtedly provoked a diminishing of species abundance. While one of the main issues when comparing these areas with different cultural traditions is the absence of precise and current data concerning the richness and abundance of mammal species, there is evidence showing that richness (and probably abundance) of species was similar throughout the Lacandon Rainforest as recently as 30 years ago [43]. However, this has varied in more recent times. While these changes lead us to think that ecological differences might be a factor influencing the cultural significance of mammals, studies evaluating the specific relation between these two variables are needed to prove it.

Several authors have described the relationships of human communities in the Neotropic region with wild fauna. They point out that mammals are considered some of the most important resources because of the use they are put to and/or the fact that people must be careful around them [65,66].

Even though the categories are the same for mestizos and Lacandons, differences are found between the two. The Lacandon population does not currently use any parts of mammas as utensils and tools, however this used to be a common use which has gradually disappeared –partly due to the integration of these communities to modern society– [67].

The main categories reported in all four communities–according to the number of species within these–are harmful, in first place, and edible, in second place. 84% of the recognized species fall into one of these categories. In general, from an *etic* perspective, ethnobiology considers that significance is based on the usefulness and use of species [68]. However, in this particular case, many mammals have a negative significance for communities because of the damage they cause to crops, cattle, or even people. This *emic* notion of mammals as harmful agents has been previously documented in the Neotropic, in particular when referring to species like *tejon* and *puerco de monte,* which damage *milpas* (maize crops) [69]. Furthermore, big carnivores are conceptualized in this negative fashion and are often eliminated because of the conflict they generate with stockbreeding communities [70]. In the case of Lacandons, these animals are kept away by setting up smoke-producing fires in each of the corners of the crop fields. Mestizos, on the other hand, use fabric softener to keep them out –this is due to the fact that, according to the interviewed, animals detect a scent that they relate to humans and keep away–, but if the animals’ presence persists, they are hunted. Among mestizos, this situation is constantly accentuated by the tendency of these communities to adopt extensive cattle breeding. Even though in Lacandon communities this species are not hunted, habitat reduction, extensive cattle breeding, and hunting in neighboring cattle breeding communities, have caused a drastic decrease of this carnivore’s population. This pattern of elimination of big cats is recurrent all across American rainforests [70,71].

The “edible” condition is, without a doubt, one of the main reasons for the conceived significance of mammals in the Neotropic region [16,41]. Consumption of wild mammals contributes an important portion of animal protein [72]. In the words of the interviewed population this use occurs basically because of the lack of resources to obtain farm animals and because of wild game. Both Lacandons and mestizos state that *carne de monte* (wild game) has a better flavor than flavor than farm animals and do not contain hormones or parasites since they are conceived as “clean animals”.

As far as the number of species related to particular CS categories differences can be appreciated even though they are not significant. Both Lacandon communities know more edible species than mestizo communities. This pattern hold for other peoples of the Neotropic region such as the Mayan in the Yucatan Peninsula [71,73]. Furthermore, among Lacandon communities there is a larger number of species included in narratives. These narratives clearly express the people’s worldview, a transcendent aspect in the understanding of their cultural traits [28]. The “narrative” category includes all tales, stories, cosmogony myths, and even the relationship of certain species to ritualistic practices. Even though some examples of narratives exist among mestizos, these are only remembered as something forefathers used to say but which is not currently believed in, particularly among the youngest. However, among Lacandons these narratives are a dynamic part of everyday life and are still transmitted to new generations [74]. Even among this indigenous group, there are cosmogony myths in which different species of mammals have a chief role in the explanation of the origin of the world and of the Lacandons (Table 4). Otherwise, mestizos conceive a greater number of species as harmful.

### Final considerations

The evidence presented in this study show that, even though ordination and classification analyses show an apparent separation among Lacandon and mestizo communities, there is no significant difference in the number of mentioned species or in the species with a high frequency of mention. Differences are found only in the least mentioned species.

It is pertinent to reflect on the reasons behind the level of importance of a given species within a community. The indicators of significance and, more importantly the causes for this valuation, respond to multifactorial processes. A greater or lesser CS can be assigned based on diverse factors, both those linked to the particular cultural features of the human group assigning it, and the intrinsic features of the species. Furthermore, these factors are shaped and re-shaped by historic processes [10,75]. In this sense, the data reported here show how different species of mammals considered significant by both mestizos and Lacandons are conceived so according to a range of factors –both positive like their different uses for the satisfaction of a series of necessities and negative, such as the harm they may cause to properties and people–.

There are some differences between mestizo and Lacandon peoples which have an influence on the way they relate to wild mammals and their general perceptions of these. It is urgent to incorporate these *emic* conceptions to preservation frameworks and strategies for mammalian species in the Lacandon Rainforest. Many of the populations in this region are abandoning the perception of the jaguar’s and other feline’s divine nature that was prevailing among ancient Mayans. Instead, these animals are currently deemed harmful agents that should be eliminated. The best we can do is characterize the dynamics underlying the conflicts between harmful species and stockbreeding populations [70] and apply diverse strategies of socio-environmental innovation privileging a dialogue of different knowledge systems and educational processes with environmental content. Contrastingly, for species such as *venado* and *tepezcuintle* are considered beneficial species, the management should be carried out mostly through strategies such as *in situ* production units. Initiatives like this are well received in rural communities and can potentially generate a mutual benefit, both for species preservation, and the revitalization of traditional cultural practices and the communities’ wellbeing.

## Abbreviations

CS: Cultural significance
MASL: Meters above sea level
APFF: Area for the protection of flora and fauna
UPGMA: Unweighted Pair Group Method with Arithmetic Mean
PCA: Principal components analysis
NTSYS: Numerical Taxonomy and Multivariate Analysis System

## References

[1] Lévi-Strauss C (1964). El pensamiento salvaje.

[2] Hunn E (1982). The utilitarian factor in folk biological classification. Am Anthropol.

[3] Bulmer R (1967). Why the Cassorwary is not a bird. Man.

[4] Douglas M (1998). Estilos de pensar.

[5] Camou A. Los recursos vegetales en una comunidad raramuri: aspectos culturales, económicos y ecológicos. Tesis doctoral. Universidad Nacional Autónoma de México, Centro de Investigaciones en Ecosistemas; 2008.

[6] Bravo-Avilés D. Relación entre la importancia cultural y atributos ecológicos en tres especies de cactáceas. Tesis de maestría. Universidad Autónoma Metropolitana; 2011.

[7] Turner NJ (1988). The Importance of a rose: evaluating the cultural significance of plants in Thompson and Lillooet interior Salish. Am Anthropol.

[8] Pieroni A (2001). Evaluation of the cultural significance of wild food botanicals traditionally consumed in northwestern Tuscany, Italy. J Ethnobiol.

[9] Turbay S, Ulloa A (2002). Aproximaciones a los estudios antropológicos sobre la relación entre el ser humano y los animales. Rostros culturales de la fauna: las relaciones entre los humanos y los animales en el contexto colombiano.

[10] Ruan-Soto F, Caballero J, Martorell C, Cifuentes J, González-Esquinca AR, Garibay-Orijel R (2013). Evaluation of the degree of mycophilia-mycophobia among Highland and lowland inhabitants from Chiapas, Mexico. J Ethnobiol Ethnomed.

[11] Heinrich MA, Ankli BF, Weimann C, Sticher O (1998). Medicinal plants in Mexico: Healers’consensus and cultural importance. Soc Sci Med.

[12] Reyes-García V, Valdez V, Tanner S, McDade T, Huanca T, Leonard WR (2006). Evaluating indices of traditional ecological knowledge: a methodological contribution. J Ethnobiol Ethnomed.

[13] Garibay-Orijel R, Caballero J, Estrada-Torres A, Cifuentes J (2007). Understanding cultural significance, the edible mushrooms case. J Ethnobiol Ethnomed.

[14] Tardio J, Pardo de Satayana M (2008). Cultural Importance Indices: a comparative analysis based on the useful wild plants of southern Cantabria (Northern Spain). Econ Bot.

[15] Alonso-Aguilar LE, Montoya A, Kong A, Estrada-Torres A, Garibay-Orijel R (2014). The cultural significance of wild mushrooms in San Mateo Huexoyucan, Tlaxcala, Mexico. J Ethnobiol Ethnomed.

[16] Monroy-Vilchis O, Cabrera L, Suárez P, Zarco-González MM, Rodríguez-Soto C, Urios V (1998). Uso tradicional de vertebrados silvestres en la Sierra Nanchititla, México. Interciencia.

[17] Londoño-Betancourth JC (2009). Valoración cultural del uso e importancia de la fauna silvestre en cautividad en tres barrios de Pereira (Risaralda). Bol Cient Mus Hist Nat.

[18] Mesquita GP, Barreto GP (2015). Evaluation of mammals hunting in indigenous and rural localities in Eastern Brazilian Amazon. Ethnobiol Conserv.

[19] Alves RRN, Souto WMS (2011). Ethnozoology in Brazil: current status and perspectives. J Ethnobiol Ethnomed.

[20] Melo RS, Silva OC, Souto A, Alves RRN, Schiel N (2014). The role of mammals in local communities living in conservation areas in the Northeast of Brazil: an ethnozoological approach. Trop Conserv Sci.

[21] Alves RRN (2012). Relationships between fauna and people and the role of ethnozoology in animal conservation. Ethnobiol Conserv.

[22] Santos-Fita D, Costa-Neto EM, Cano-Contreras EJ, Costa-Neto E, Santos-Fita D, Vargas-Clavijo M (2009). El quehacer de la etnozoología. Manual de Etnozoología.

[23] Albuquerque UP, Lucena RFP, Monteiro JM, Florentino ATN, Almeida CFCBR (2006). Evaluating two quantitative ethnobotanical techniques. Ethnobot Res Appl.

[24] Montoya A, Torres-García E, Kong A, Estrada-Torres A, Caballero J (2012). Gender differences and regionalization of the cultural significance of wild mushrooms around La Malinche Volcano, Tlaxcala, México. Mycologia.

[25] Hilgert N, Contreras-Ramos A, Cuevas Cardona C, Goyenenchea I, Iturbe U (2007). La Etnobotánica como herramienta para el estudio de los sistemas de clasificación tradicionales. La Sistemática, base para el conocimiento de la biodiversidad.

[26] Thompson E, Juan Z (2006). Comparative cultural salience: measuring using free list data. Field Method.

[27] Phillips O, Alexiades M (1996). Some quantitative methods for analyzing ethnobotanical knowledge. Selected guidelines for ethnobotanical research: a field manual.

[28] Broda J, Báez-Jorge F (2001). Cosmovisión, ritual e identidad de los pueblos indígenas de México.

[29] García-Gil JG, Lugo J, Vázquez-Sánchez MA, Ramos MA (1992). Las formas del relieve y los tipos de vegetación en la Selva Lacandona. Reserva de la Biosfera Montes Azules, Selva Lacandona: Investigación para su conservación.

[30] INEGI: Censo de población y vivienda 2010. Instituto Nacional de Estadística Geografía e Informática. México D.F. [http://www.inegi.org.mx/]

[31] Alexiades MN (1996). Selected guidelines for ethnobotanical research: a field manual.

[32] Weller SC, Romney AK (1988). Systematic data collection.

[33] Sandoval C (2002). Investigación cualitativa. Programa de especialización teórica, métodos y técnicas de investigación social.

[34] Rohlf FJ (2005). NTSYS-pc: Numerical Taxonomy and Multivariate Analysis System, Version 2.2.

[35] Berlin B, Breedlove D, Raven P (1973). General principles of classification and nomenclature in folk biology. Am Anthopol.

[36] Lorenzo C, Cruz L, Naranjo E, Barragán F (2007). Uso y conservación de mamíferos silvestres en una comunidad de las Cañadas de la Selva Lacandona, Chiapas, México. Etnobiologia.

[37] Guerra M. Cacería de subsistencia de dos localidades de la Selva Lacandona, Chiapas, México. Tesis de licenciatura. Universidad Nacional Autónoma de México, Facultad de Ciencias; 2001.

[38] Cossío A. Conocimiento y comparación del uso de la fauna silvestre en dos comunidades ejidales del municipio de Hueytamalco, Puebla, México. Tesis de Maestría. Instituto de Ecología; 2007.

[39] Vélez SD, Wildlife Conservation Society (2004). Diagnóstico del uso de fauna silvestre en las veredas mundo nuevo, el Manzano y la Jangada en la Reserva Forestal Protectora de los Ríos Blanco y Negro en el Municipio de la Calera (Cundinamarca -Colombia). Memorias del VI Congreso Internacional sobre Manejo de Fauna Silvestre en la Amazonía y Latinoamérica: *5*–*10 September 2004*; Iquitos.

[40] Tejada R, Chao E, Gómez H, Painter L, Wallace B (2006). Evaluación sobre el uso de fauna silvestre en la tierra comunitaria de origen Tacana, Bolivia. Ecol Boliv.

[41] Castro C. Diagnóstico socioambiental del uso de fauna silvestre en el Bosque Protector Alto Nangaritza-Región Sur del Ecuador. Tesis de Licenciatura. Universidad Técnica Particular de Loja; 2008.

[42] Cunha RG, Schiavetti A, Costa-Neto E, Santos-Fita D, Vargas-Clavijo M (2009). Conocimiento, creencias y utilización de la mastofauna por los pobladores del Parque Estatal de la Sierra de Conduru, Bahia, Brasil. Manual de Etnozoología.

[43] Álvarez del Toro M (1977). Los mamíferos de Chiapas.

[44] Bisbal E (1994). Consumo de fauna silvestre en la zona de Imataca, Estado Bolivar, Venezuela. Interciencia.

[45] Escamilla A, Sanvicente M, Sosa M, Galindo-Leal C (2000). Habitat mosaic, wildlife availability, and hunting in the tropical forest of Calakmul, México. Cons Biol.

[46] Rumiz D, Maglianesi M (2001). Hunting impacts associated whit Brazil nut harvesting in the Bolivian Amazon. Vida Silvestre Neotrop.

[47] Barbarán FR (2003). Factibilidad de caza de subsistencia, comercial y deportiva en el Chaco semiárido de la Provincia de Salta, Argentina. FERMENTUM Mérida.

[48] Rodríguez A, Van Der Hammen M, Cites and Fundación Natura (2003). Manejo indígena de la fauna en el medio y bajo rio Caquetá (Amazonia colombiana). Tradición, transformaciones y desafíos para su uso sostenible. Manejo de Fauna Silvestre en Amazonía y Latinoamérica: selección de trabajos V Congreso Internacional de Manejo de fauna silvestre en Amazonia y Latinoamérica. 10–14 September 2001; Cartagena de Indias.

[49] Naranjo EJ, Guerra M, Bodmer R, Bolaños J (2004). Subsistence hunting by three ethnic groups of the lacandon forest, Mexico. J Ethnobiol.

[50] Lira TI (2006). Abundancia, densidad, preferencia de hábitat y uso local de los vertebrados en la tuza de Monroy Santiago Jamiltepec, Oaxaca. Rev Mex Mastozool.

[51] Cordeiro RK, Drumon P (2007). Caracterização da caça de subsistência em dois seringais localizados no Estado do Acre (Amazônia, Brasil). Embrapa Acre.

[52] Racero-Casarrubia C, Vidal C, Ruiz O, Ballesteros J (2008). Percepción y patrones de uso de la fauna silvestre por las comunidades indígenas Embera-Katíos en la cuenca de rio San Jorge, zona amortiguadora del PNN-Paramillo. Rev Estud Soc.

[53] Rosales MM, Hermes MS, Morales JR, Guerra M, Calmé S, Gallina S, Naranjo EJ (2010). Caracterización de la cacería de subsistencia en comunidades Maya-Q’eqchi’ del área de influencia del Parque Nacional Laguna Lachuá, Guatemala. Uso y manejo de la fauna silvestre en el norte de Mesoamérica.

[54] Aquino RC, Terrones C, Navarro R, Terrones W (2007). Evaluación del impacto de la caza en mamíferos de la cuenca del rio Alto Itaya, Amazonia peruana. Rev Perú Biol.

[55] Cuesta-Rios EY, Valencia-Mazo J, Jiménez-Ortega J (2007). Aprovechamiento de los vertebrados terrestres por una comunidad humana en bosque tropicales (Tutunendo, Chocó, Colombia). Rev Inst Univ Tec Chocó: Invest Biodiv Des.

[56] Méndez-Cabrera F, Montiel S (2007). Diagnostico preliminar de la fauna y flora silvestre utilizada por la población Maya de dos comunidades costeras de Campeche, México. Univ Ciencia.

[57] Sánchez A, Vásquez P (2007). Presión de caza de la comunidad nativa Mushuckllacta de Chipaota, zona de amortiguamiento del Parque Nacional Cordillera Azul, Perú. Ecol Apl.

[58] Centeno P, Arriaga S, Guerra M, Calmé S, Gallina S, Naranjo EJ (2010). Uso y aprovechamiento de fauna silvestre en comunidades del Parque Estatal de La Sierra, Tabasco, México. Uso y manejo de la fauna silvestre en el norte de Mesoamérica.

[59] Read J, Fragoso J, Silvius M, Luzar J, Overman H, Cummings A (2010). Space, place, and hunting patterns among indigenous peoples of the Guyanese Rupununi Region. J Lat Am Geography.

[60] Matos E, Catálogo de la exposición (2003). Mesoamérica antigua. Iberoamérica mestiza, encuentro de pueblos y culturas.

[61] Rabinowitz RA (1986). Jaguar predation on domestic livestock in Belice. Wildlife UIHUI Soc Bull.

[62] Perovic P, Herrán M (1998). Distribución del jaguar *Panthera onca* en las provincias de Jujuy y Salta, noroeste de Argentina. Mastozool Neotrop.

[63] Miller B, Rabinowitz A, Medellín R, Equihua C, Chetkiewicz CH, Crawshaw P, Rabinowitz A, Redford K, Robinson J, Sanderson E, Taber A (2002). ¿Por qué conservar al jaguar?. In *El Jaguar en el nuevo milenio*.

[64] Scognamillo D, Maxit E, Sunquist M, Farell L, Medellín R, Equihua C, Chetkiewicz CH, Crawshaw P, Rabinowitz A, Redford K, Robinson J, Sanderson E, Taber A (2002). Ecología del jaguar y el problema de la depredación de ganado en un hato de los llanos Venezolanos. El Jaguar en el nuevo milenio.

[65] Ojasti J (1993). Utilización de la fauna silvestre en América Latina situación y perspectivas para un manejo sostenible. Guía FAO conservación 25.

[66] Carr HS, Fedick SL (1996). Precolumbian maya exploitation and management of deer populations. The managed mosaic: ancient maya agriculture and resource use.

[67] Toledo V, Ortiz-Espejel B, Cortés L, Moguel P, Ordoñez MDJ (2003). The multiple use of tropical forests by indigenous peoples in Mexico: a case of adaptive management. Cons Ecol.

[68] Phillips OL, Gentry AH (1993). The useful plants of Tambopata Peru: I: statistical hypotheses tests with a new quantitative technique. Econ Bot.

[69] Romero-Balderas K, Naranjo EJ, Morales HH, Nigh RB (2006). Daños ocasionados por vertebrados silvestres al cultivo de maíz en la selva lacandona, Chiapas, México. Interciencia.

[70] Schulz F, Printes RC, Oliveira LR (2014). Depredation of domestic herds by pumas based on farmer’s information in Southern Brazil. J Etnobiol Ethnomed.

[71] Santos-Fita D, Naranjo EJ, Rangel-Salazar JL (2012). Wildlife uses and hunting patterns in rural communities of the Yucatan Peninsula, Mexico. J Etnobiol Ethnomed.

[72] Redford KH, Robinson JG (1987). The game of choice: patterns of Indian and colonist hunting in the neotropics. Am Anthropol.

[73] Quijano-Hernández E, Calmé S (2002). Patrones de cacería y conservación de la fauna silvestre en una comunidad maya de Quintana Roo, México. Etnobiología.

[74] Cano Contreras E, Erosa E, Mariaca R (2009). Tu chien k’an. Un recorrido por la cosmovisión de los lacandones del Norte desde las mordeduras de serpiente.

[75] Pagaza-Calderón EM, González-Insuasti MS, Pacheco-Olvera RM, Pulido MT (2006). Importancia cultural, en función del uso, de cinco especies de artrópodos en Tlacuilotepec, Puebla, México. Sitientibus Sér Ciênc Biol 6 (Etnobiologia).

